# Transfer learning based generalized framework for state of health estimation of Li-ion cells

**DOI:** 10.1038/s41598-022-16692-4

**Published:** 2022-08-01

**Authors:** Subhasmita Sahoo, Krishnan S. Hariharan, Samarth Agarwal, Subramanian B. Swernath, Roshan Bharti, Seongho Han, Sangheon Lee

**Affiliations:** 1 Samsung R&D Institute India-Bangalore, Bangalore, 560037 India; 2 Advanced Lab. - Battery, SAMSUNG Electronics, Suwon, Gyeonggi-do 16677 Republic of Korea

**Keywords:** Electrical and electronic engineering, Batteries

## Abstract

Estimating the state of health (SOH) of batteries powering electronic devices in real-time while in use is a necessity. The applicability of most of the existing methods is limited to the datasets that are used to train the models. In this work, we propose a generic method for SOH estimation with much wider applicability. The key problem is the identification of the right feature set which is derived from measurable voltage signals. In this work, relative rise in voltage drop across cell resistance with aging has been used as the feature. A base artificial neural network (ANN) model has been used to map the generic relation between voltage and SOH. The base ANN model has been trained using limited battery data. Blind testing has been done on long cycle in-house data and publicly available datasets. In-house data included both laboratory and on-device data generated using various charge profiles. Transfer learning has been used for public datasets as those batteries have different physical dimensions and cell chemistry. The mean absolute error in SOH estimation is well within 2% for all test cases. The model is robust across scenarios such as cell variability, charge profile difference, and limited variation in temperature.

## Introduction

An increasing number of electronic devices such as electric vehicles and mobile phones rely on rechargeable batteries as the only source of power for operation. As a battery is repeatedly charged and discharged, known as cycling, the available capacity decreases continuously thereby degrading the battery. Hence, every battery traverses through a health trajectory starting from a perfectly healthy state to a completely dead state. Accurate, on-device estimation of battery state of health (SOH) is essential to monitor the battery condition. The algorithm should be robust enough to be compatible with rapidly evolving battery specifications. SOH of a battery is defined as the relative change in capacity over the charge-discharge cycles. For example, SOH of a battery at the $n$th cycle can be defined as

1 $$\begin{aligned} SOH_n = \dfrac{C_n}{C_{rated}}; \end{aligned}$$

where, $C_{rated}$ is rated/nominal capacity of the battery, and $C_n$ is the capacity of the battery after *n* number of cycles. SOH has to be estimated from the measured voltage and current signals.

State-of-the-art SOH estimation algorithms can be broadly categorized into model-based methods and data-driven methods^1^. Model-based techniques use estimation algorithms such as Kalman filter^2,3^, particle filter^4,5^, etc. to estimate SOH from equivalent circuit model of the battery. Data-driven methods use regular charge or discharge data and corresponding SOH to train machine learning models^1,6^. With time, data-driven methods have gained popularity due to ease of use, and advancement in the computational capability of machines. Further, the potential of data-driven methods can be enhanced as more data becomes available. This ensures improved accuracy with minimal intervention in the underlying computation.

Another set of SOH estimation methods rely on the shift in peaks from an incremental capacity (IC) analysis. A shift in peak height in the IC curve (*dQ*/*dV* vs *V* plot) can be observed with a decrease in SOH. This information has been exploited in^7–10^ for SOH estimation. These methods have been trained and tested using data of the same battery. Other IC curve features such as peak height, peak voltage, and peak area have been used in^11^ and^12^ for SOH estimation. Though the authors have used exclusive test battery data for model evaluation, effect of charge profile variation on the algorithm performance has not been explored. Long-short term memory (LSTM) based SOH prediction algorithm in^1^ and LSTM+ANN based algorithm proposed in^13^ also use initial cycle data of the test battery for training. The robustness of these algorithms to varying battery specifications and charge profiles has not been reported.

Change in state of charge (SOC) vs open circuit voltage (OCV) curve with aging has been utilized in^14^ for the estimation of capacity degradation. In^15^, the shift in charging voltage curve due to aging has been used as feature to train support vector machine (SVM) models. Unlike most of the state-of-the-art methods, the relation between battery surface differential temperature and SOH has been used to train a support vector regression model in^16^. All these methods have been tested on limited cell data, and their robustness to changes in battery type and charging profile has not been investigated. Constant current (CC) charge time and time to charge between two voltage levels reduce with aging. In^17^, these charge-time-based parameters have been used as input to the least square SVM for SOH estimation. However, any variation in charging current profile would change the elapsed time features thereby deviating the output of SVM.

Incremental voltage difference has been used as a feature in^18^ to train a shallow ANN. However, as simulated data has been used for training and the slope of voltage curve has been used as a feature, the method might fail if charge profile or device specifications change. The energy of equal discharge voltage difference has been used in^19^ as a health indicator (HI). Features extracted from the smoothed HI curve have been used in an ANN to estimate SOH. Transfer learning with convolutional neural networks (CNN) has been used in^20^. The CNN trained using accelerated aging data of cells has been fine-tuned on 15% data of unknown cells, and the rest 85% data has been used to test the algorithm. Though the methods proposed in^19^ and^20^ seem promising, their efficacy on data with different charge profile (other than CCCV such as multi-step CCCV (MSCCCV)) have not been explored. Transfer learning with neural network models have been used in^13,20–22^ for SOH estimation. However, they use transfer learning to fine-tune the offline trained models on the data of target batteries. Though, it eliminates training, but requires parameter tuning for every new cell.

Existing SOH estimation algorithms are effective in estimating SOH when the variation across battery type (battery specifications such as capacity, internal resistance, etc.) and charge profile (CCCV or MSCCCV charging) is assumed to be the same in training and testing. To the best of our knowledge, none of the methods address the challenges associated with variation in charge profile and battery specification. The existing SOH estimation methods lack extensive testing on different batteries. Most of those methods would require model tuning to estimate the SOH of an unseen cell.

To overcome these issues, the feature has to be robust. It has to be unaffected by variations in battery behavior and charge profiles. It would not require re-training or fine-tuning of SOH estimation models for every new battery. From the analysis of several Li-ion cell data, it has been observed that the voltage drop across cell resistance increases proportionately with degradation in capacity. The absolute values of voltage drop and SOH may differ for similar batteries; however, the relative change largely remains the same. Therefore, the relative increase in voltage drop with aging has been used as a feature to estimate the relative drop in battery SOH. The relation between feature and SOH is independent of C-rate and charge profile. Following are the contributions made in this paper.

1. A novel feature has been proposed which is independent of operating conditions such as charging current profile (CCCV charging or MSCCCV charging etc.), C-rate, and limited variation in temperature.
2. A relative quantity instead of absolute values (absolute quantities might change with battery variability), obtained directly from measured quantities, has been used as feature to estimate the relative battery degradation. The relative increase in voltage drop over cycles has been mapped to the relative decrease in SOH.
3. The model is trained or fine-tuned using only one cell data of a particular chemistry and physical dimension. After that, it can be used for other cells irrespective of charge profile, C-rate, and limited variation in temperature.
4. Extensive testing of the proposed method has been done using both offline and online battery data having different charge profile, capacity, and operating temperature. The SOH estimation error remains low in all test scenarios.

**Figure 1:**
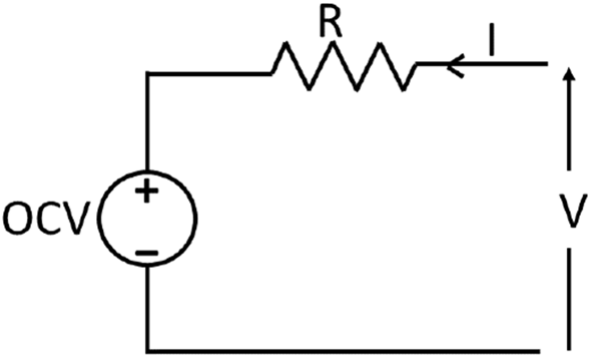
Electrical equivalent circuit of battery.

**Figure 2:**
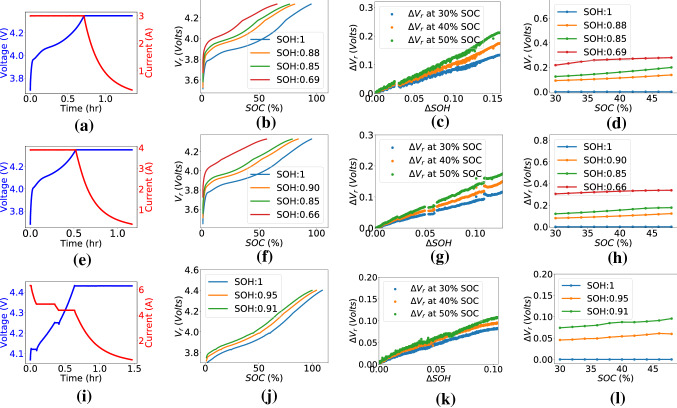
(**a**), (**e**), (**i**) Voltage current plots for charge profile 1C-CCCV, 1.3C-CCCV, 1C-MSCCCV respectively. (**b**), (**f**), (**j**) $V_r$ at different SOH values for charge profiles in (**a**), (**e**), (**i**) respectively. (**c**), (**g**), (**k**) Correlation between $\Delta V_r$ and $\Delta SOH$ for plots in (**b**), (**f**), (**j**) at 3 different SOC points. (**d**), (**h**), (**l**) A set of features computed from the plots in (**b**), (**f**), (**j**) respectively.

## Results

### Feature selection

The robustness and accuracy of SOH estimation largely depend on the effectiveness of extracted features. A thorough study of literature indicates that SOH features lack robustness to change in battery operating conditions such as current rate, charging current profile, temperature, etc. For example, time stamp to charge a battery between two voltage intervals^13^ will change if the C-rate or charge profile changes. The slope of the voltage curve used in^18^ varies with SOH in the CC region. The slope variation will be negligible in the CV region, and thus would be inefficient if the charge profile changes to MSCCCV or to a CCCV profile with small CC region. A robust feature to estimate SOH from the measured voltage and current has been the need of the hour.

In order to select a feature for SOH estimation, laboratory cycled data was analyzed. A resistance model (Fig. 1) was assumed as the electrical equivalent circuit of the battery^23^. In the figure, *R* is the resistance, and OCV is the open-circuit voltage of the battery. *V* and *I* are charging voltage and current respectively. It was observed from the data that voltage drop across the resistance was increasing with aging. The quantity reflecting incremental voltage drop was computed by subtracting the drop across fresh cell resistance from the measured voltage. An average fresh cell resistance of 60 m$\Omega$ was assumed as the measured resistance of fresh cells were in the range of 50–70 m$\Omega$. The fresh cell resistance was computed using voltage jump at the beginning of charge when step charging current was applied.

**Figure 3:**
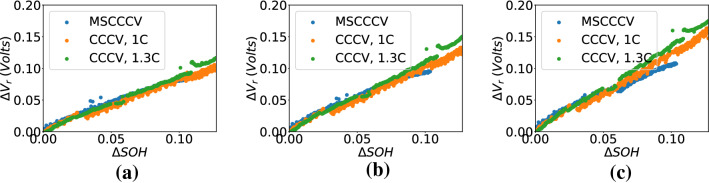
$\Delta V_r$ vs $\Delta SOH$ plot for different charge profiles at (**a**) 30% SOC, (**b**) 40% SOC, (**c**) 50% SOC.

Let $V_r$ be the quantity reflecting the increase in voltage drop across battery resistance. $V_r$ is computed by subtracting the voltage drop across the fresh battery resistance (cycle 0) from the measured terminal voltage. In other words, $V_r$ represents the combined voltage of cell OCV and the incremental voltage drop across the resistance.

2 $$\begin{aligned} V_r = V-I*R_0, \end{aligned}$$

where $R_0$ is the resistance of the fresh battery, *V* is the measured voltage, and *I* is the charging current. *SOC*(*t*) (%) at an instant *t* after beginning of charge was computed using current and sampling interval *dt*

3 $$\begin{aligned} SOC(t)(\%) = SOC(0)(\%)+\frac{\int _{0}^{t} Idt}{C_{rated}}\times 100, \end{aligned}$$

where $t=0$ refers to the beginning of charge in a cycle, and $C_{rated}$ is the rated capacity of the battery. $V_r$ at a fixed *SOC* was found to be increasing with battery aging. Figure 2 shows feature plots for three different charging protocols: (a)1C-CCCV, (e)1.3C-CCCV, and (i)1C-MSCCCV. $V_r$ vs *SOC* at different SOH values for these 3 charging protocols have been plotted in Fig. 2b,f, and j respectively. A clear shift in $V_r$ vs *SOC* curves can be observed with a decrease in SOH in all three cases. Value of $V_r$ might get affected by differences in voltage and current profile and $R_0$ value; whereas, the relative shift in $V_r$ has been observed to be similar across batteries. Therefore, unlike^18^, the relative change in $V_r$ over cycles has been selected as a feature. Let the relative shift in $V_r$ vs *SOC* curve with respect to that at $SOH=1$ (cycle 0 or fresh battery) be denoted as $\Delta V_r$.

4 $$\begin{aligned} \{\Delta V_{r_c}\}_{SOC_i} = \{V_{r_c}\}_{SOC_i} - \{V_{r_0}\}_{SOC_i}, \end{aligned}$$

where $20\%<SOC_i<90\%$. $V_{r_c}$ and $V_{r_0}$ are $V_r$ of cycle *c* and 0 respectively. $\Delta V_{r_c}$ represents $\Delta V_r$ for cycle *c*. $\Delta V_r$ is computed by sampling $V_{r_c}$ and $V_{r_0}$ based on *SOC* values. The advantage of using $\Delta V_{r}$ as feature is that any component of $V_r$ which remains constant with aging will be canceled in the $\Delta V_{r}$ computation, and any other voltage component which varies with aging will be reflected in the feature. Let $\Delta SOH$ be the drop in SOH (from 1) corresponding to $\Delta V_r$ shift in $V_r$. $\Delta V_r$ at 30%, 40%, and 50% SOC have been plotted against $\Delta SOH$ in (c), (g), and (k) respectively for charge profiles in (a), (e), and (i) of Fig. 2. $\Delta V_r$ can be seen to be almost linearly varying with $\Delta SOH$ at fixed *SOC* points. $\Delta V_r$ vs $\Delta SOH$ for all 3 charge profiles have been shown in a single plot at two different SOC points in Fig. 3. It can be observed that the feature-label proportionality does not change with variation in charge profile.

### SOH estimation of exclusive test battery set

A base ANN model was trained using data from 8 cells which makes about 1/4 of the total number of batteries involved in experiments. Input to the ANN is $\Delta V_r$ features computed from charging voltage and current. $\Delta SOH$ is the output of the ANN. The same base ANN has been used in all tests described hereafter, without any further training. Batteries which were not involved in training of the base ANN were used for blind testing of the proposed algorithm. Blind testing was performed to confirm the robustness of the trained base ANN to cell variability. The method was tested on long cycled battery data to validate its performance in advanced cycles. For quantitative evaluation, the estimated SOH values were compared against the ground truth SOH computed from low current (0.2C) probe cycles.

#### Robustness to charge profile variability

**Figure 4:**
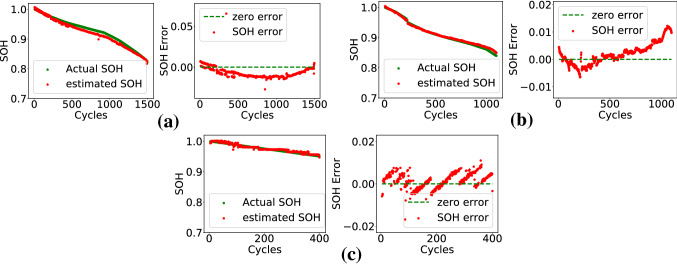
Plots of actual and estimated SOH, and error for laboratory testing. (**a**) S1-B3: 0.8C, CCCV charging, (**b**) S1-B6: 1.2C, CCCV charging, and (**c**) S6-B1: 1C MSCCCV charging.

The base ANN was tested on exclusive test battery data. Different charging protocols had been used in cycling of these batteries. Test accuracy for in-house laboratory generated data have been given in Table 1 in the form of mean absolute error (MAE), root mean square error (RMSE), and standard deviation error (SDE). MAE is well within the range of 0.02, and it is below 0.01 for most of the cases. The error is not affected by variation in charging C-rates for S1 batteries. Also, the MAE values for both CCCV and MSCCCV charge profiles are within 0.02. Estimated SOH and error of two S1 cells charged using 0.8C CCCV (B3) and 1.2C CCCV (B6) profiles have been plotted in Fig. 4a,b respectively. Figure 4c shows the estimated SOH and error plot for S6-B1 which had been charged using 1C MSCCCV profiles. It can be observed that the estimated SOH is close to the ground truth even in the advanced cycles.

**Table 1:** SOH estimation results for laboratory data test.

Test battery	MAE	RMSE	SDE
S1-B1	0.0045	0.005	0.0046
S1-B2	0.0084	0.0092	0.006
S1-B3	0.0087	0.01	0.0083
S1-B4	0.0153	0.0162	0.006
S1-B5	0.0121	0.0199	0.0158
S1-B6	0.0055	0.0122	0.0141
S1-B7	0.0081	0.021	0.0207
S2-B1	0.0118	0.0133	0.0064
S2-B2	0.0027	0.0079	0.0034
S2-B3	0.0061	0.007	0.0035
S2-B4	0.0036	0.0043	0.004
S5-B1	0.0044	0.0069	0.0066
S6-B1	0.0032	0.0039	0.0035
S7-B1	0.0032	0.0044	0.0042
S8-B1	0.0041	0.0048	0.004

#### Validation in the presence of noise

**Figure 5:**
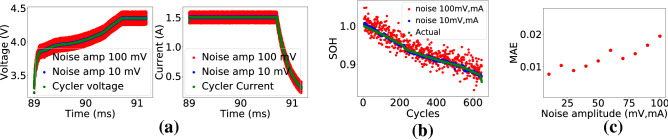
SOH estimation in the presence of noise. (**a**) Voltage and current with added noise, (**b**) Estimated SOH in the presence of noise, (**c**) SOH estimation MAE at different noise levels.

On-device measured voltage and current have inherent noise due to associated measurement uncertainty. The effect of the noise on performance of the proposed method has been validated by adding random noise to the cycler data. The measurement uncertainty of the devices used in the experiment are in the range of ţV and ţA. The added noise amplitude was increased beyond the ţV and ţA range to measure the noise tolerance limit of the method (assuming an acceptable SOH estimation MAE 2%). Amplitude of the noise added to the cycler voltage and current were increased in steps of 10 mV and 10 mA starting from 1 mV and 1 mA respectively. The SOH estimation method was applied to the noisy data. The results indicated that the MAE hit the tolerance limit of 2% at a noise level around 100 mV, mA. The MAE vs noise amplitude plot, estimated SOH, and noisy voltage and current plots have been shown in Fig. 5. It is evident from the plots that the MAE increases with increase in noise level. If an MAE tolerance limit is set at 2%, then the method can be said to be robust to noise of 100mV and 100mA in voltage and current measurements respectively.

### SOH estimation of on-device batteries

Charging data of batteries in a laboratory environment are devoid of device noise. However, data acquired from on-device batteries are vulnerable to noise because of several factors such as temperature, device usage, etc. Therefore, to test the real-time applicability of the method, it was tested on some of the batteries mounted on user devices.

#### Robustness to on-device noise

**Figure 6:**
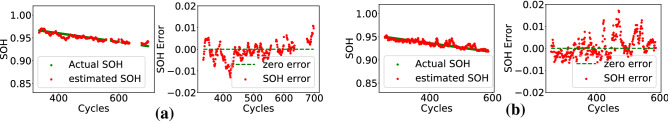
Plots of actual and estimated SOH, and error for on-device testing. (**a**) S2-B1, (**b**) S4-B1.

The same base ANN was used to estimate SOH of batteries mounted on devices. An android based application was made which ran the proposed algorithm in the background while batteries were charged and discharged using device-specific chargers. Assuming that identical batteries will age at the same rate, SOH computed from laboratory cycled data of similar batteries were used as the ground truth for on-device experiments. The accuracy numbers have been shown in Table 2. MAE values are in the same range as laboratory test results. Estimated SOH and error plots of two batteries from the S1 and S2 set have been shown in Fig. 6a,b respectively.

**Table 2:** SOH estimation results for on-device test.

Test battery	MAE	RMSE	SDE
S2-B1	0.0068	0.01	0.0095
S2-B2	0.0066	0.0094	0.008
S2-B3	0.0051	0.0056	0.0041
S2-B4	0.0036	0.0041	0.0024
S3-B1	0.0122	0.0163	0.0111
S4-B1	0.0034	0.0045	0.0044
S4-B2	0.0011	0.0014	0.0009
S4-B3	0.0017	0.0019	0.0007
S5-B1	0.0066	0.0092	0.0074
S5-B2	0.0094	0.0116	0.0076
S5-B3	0.0033	0.0034	0.0009
S5-B4	0.0031	0.0032	0.00076
S7-B1	0.0109	0.011	0.0011
S8-B1	0.0184	0.0198	0.0072

### Transfer learning for unseen dataset

As the base ANN was trained using data from multiple batteries, it is expected to work for batteries with similar $\Delta V_r$ vs $\Delta SOH$ relation (Fig. 3). However, if the battery type or physical dimension of another set of batteries are significantly different, then the feature-label proportionality relation might change^24,25^. In such a scenario, transfer learning was used. The first fully connected layer in the base ANN extracts information from input features; the second layer maps the proportionality relation between input and output. Therefore, the first layer of the base ANN (trained using in-house data) was kept unchanged, and only the second layer was fine-tuned on single-cell data of the new set. Hyper parameters such as loss function, optimizer, etc. was the same as that used for training of the base ANN.

#### Generalization to different battery datasets

The proposed method was tested on two publicly available datasets: CALCE^26,27^, and NASA^28^. The batteries used in these data were of different physical dimension and cell chemistry than in-house batteries used for training of the base ANN. Therefore, the feature-SOH relation will be different and the base ANN will not work for these datasets. Transfer learning was used to make the base ANN work for the new data. The last fully-connected layer of the base ANN was fine-tuned using one randomly chosen cell data from each dataset. The rest of the batteries in the set were used for testing.

**Figure 7:**
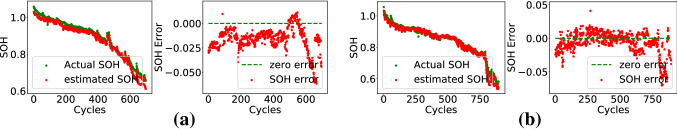
Plots of actual and estimated SOH, and error for (**a**) battery CS2-34, (**b**) battery CS2-38 of CALCE dataset.

CS2 batteries of CALCE data were used in the experiment. Fine-tuning of ANN was done using CS2-33 data. CS2-34, 35, 36, 37, 38 were used for blind testing. The accuracy numbers have been given in Table 3. The estimated SOH and error in estimation have been plotted in Fig. 7 for two batteries in the set. The MAE is within 0.02 for all the cases.

**Table 3:** SOH estimation results for CALCE data.

Test battery	MAE	RMSE	SDE
CS2-34	0.0168	0.0199	0.0118
CS2-35	0.0121	0.0157	0.0119
CS2-36	0.0132	0.0158	0.0091
CS2-37	0.0084	0.0117	0.0103
CS2-38	0.0114	0.0169	0.0157

**Table 4:** SOH estimation results for NASA data.

Temperature	Test battery	MAE	RMSE	SDE
Room Temp	B06	0.0103	0.0127	0.0126
	B07	0.01	0.0113	0.0064
	B18	0.0324	0.0352	0.0157
43$^{\circ }$ C	B29	0.0034	0.0041	0.004
	B30	0.0089	0.0102	0.006
	B31	0.0036	0.0045	0.0043
	B32	0.0167	0.0194	0.0099

**Figure 8:**
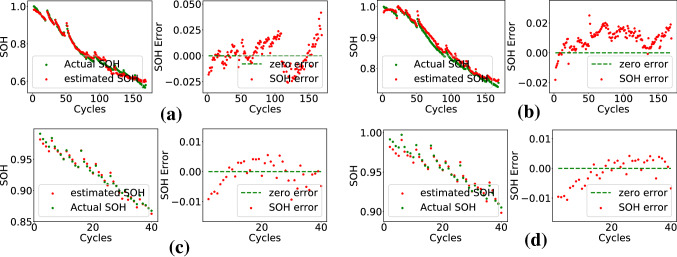
Plots of actual and estimated SOH, and error (**a**) B06, (**b**) B07, (**c**) B29, (**d**) B31 of NASA dataset.

Battery data collected at two different temperatures were used in the experiment. Data of B05, B06, B07, and B18 were collected at room temperature. B29, B30, B31, and B32 data were collected at an elevated temperature of 43$^{\circ }$ C. The last FC layer of the base ANN was fine-tuned on B05 data. Other cell data were used for blind testing. The accuracy numbers have been given in Table 4. The estimated SOH and error in estimation have been plotted in Fig. 8 for two batteries in the set. The MAE is within 0.02 for most of the cases except B18. It can also be observed that the feature-SOH relation is not getting affected by the variation in temperature. SOH estimation accuracy of the proposed method on CALCE and NASA data have been compared with that reported in two recently published methods^13^ and^29^. The comparison has been shown in Table 5. The accuracy figures for the proposed method are comparable and in some cases better than that of state-of-the-art. Additionally, this method does not require ANN fine-tuning for every cell. It is evident that fine-tuning of only second layer of the base ANN (trained using in-house data) on one cell data of the new dataset is sufficient to test on other batteries in the dataset. The SOH estimation method reported in^13^ uses LSTM for SOH computation. It can be seen from Table 5 that the proposed technique has lower SDE in most of the cases as compared^13^. The better accuracy can be attributed to the feature as the neural network is much simpler as compared to the LSTM model.

**Table 5:** Performance comparison with state-of-the-art methods.

Battery no	Proposed Method			^13^		^29^
	MAE	RMSE	SDE	RMSE	SDE	MAE
CS2-35	0.0121	0.0157	0.0119	0.0052	0.063	
CS2-36	0.0132	0.0158	0.0091	0.0104	0.3233	
CS2-37	0.0084	0.0117	0.0103	0.0075	0.09	
CS2-38	0.0114	0.0169	0.0157	0.0073	0.0525	
B05	0.0076	0.0103	0.0092	0.0054	0.0398	0.0119
B06	0.0103	0.0127	0.0126	0.0143	0.0412	
B07	0.01	0.0113	0.0064	0.0049	0.079	0.0128
B18	0.0324	0.0352	0.0157			0.0288

## Discussion

Good the feature is, robust will be the SOH estimation. In this work, a novel, efficient, and generic feature, computed solely from the measurable voltage and current, has been proposed. The feature has a proportional relation with battery SOH. Also, the feature-SOH proportionality relation has been observed to be independent of the charge profile, C-rate, and limited variation in temperature. The voltage drop across the series resistance has been observed to be increasing with the drop in battery SOH. As a result, the voltage across the resistance vs SOC curve shifts upwards. The relative voltage shift has been seen to be independent of charge profile (CCCV or MSCCCV) and C-rate. A two-layer ANN has been used to map the variation in voltage into SOH.

The proportionality relation between feature and label is the same for similar types of batteries, i.e. batteries having similar physical dimension and base chemistry. Hence, the base ANN can be used to estimate the SOH of similar batteries at an MAE within 0.02. However, the proportionality relation might be different for a battery set having different physical dimension and chemistry than the training set. In that scenario, fine-tuning of the final fully connected layer of ANN has been done for SOH estimation. The use of only one battery data for fine-tuning has been observed to be sufficient for SOH estimation of other batteries with an MAE limit of 0.02.

The proposed method has been verified using 37 in-house battery data The batteries were cycled in laboratory and user devices using different charge profiles and C-rates. Test on the exclusive battery set resulted in SOH estimation MAE within 0.02. The method has also been tested on two publicly available battery datasets: CALCE and NASA. SOH estimation error for almost all the batteries of the two datasets was within 0.02. Test on NASA battery data cycled at room temperature and 43 °C confirmed the robustness of the proposed method to temperature variation. Following are the advantages of the proposed method compared to deep learning methods.

1. It involves less computation as network is shallow. It can be used on edge devices for real time battery health monitoring.
2. It is a generic method. The model does not require retraining or fine-tuning for every new cell. Transfer learning is done only once when the battery chemistry and dimension changes.
3. The method is robust to variation in charge profile, C-rate, and temperature.

## Methods

This section describes the SOH estimation technique. The experimental data generation process has been explained in detail, followed by feature selection criteria. The feature has been selected based on the analysis of experimentally generated data. The ANN used for SOH mapping has been described next. Steps to estimate SOH and the evaluation metrics have been outlined at the end of this section.

### Experimental data generation

Multiple cells of different capacity and charge protocols as listed in Table 6 were used in the experiment. The basic chemistry for all these batteries was the same. It was an LCO cathode- Graphite anode pouch cell configuration. Depending on the manufacturer, there are variations in battery raw material, manufacturing process, additives, etc. As a result, the behavior of batteries such as resistance magnitude and SOC-OCV profile changes. To generate data for algorithm development and validation, 23 batteries were cycled in the laboratory. The batteries were charged and discharged repeatedly while allowing it to rest (current = 0) for 10 min between every charge and discharge. Current, voltage, and timestamps were recorded during the cycling process. For real-time validation of the algorithm, 14 batteries were cycled in commercial devices by replicating user charge-discharge scenario. Battery specifications such as capacity and charge protocol have also been indicated in Table 6. All S1 batteries have been cycled at different current rates (0.6C, 0.7C, 0.8C, 0.9C, 1C, 1.1C, 1.2C, 1.3C).

**Table 6:** Batteries used in experiments.

Device set no.	Max capacity (Ah)	Cycled in laboratory		Cycled on device	
		No. of batteries	Charge protocol	No. of batteries	Charge protocol
S1	3.0	8	CCCV	0	–
S2	3.89	5	MSCCCV	4	CCCV
S3	4.37	1	MSCCCV	1	CCCV
S4	4.37	1	MSCCCV	3	CCCV
S5	5.83	2	CCCV	4	CCCV
S6	3.88	2	MSCCCV	0	–
S7	4.37	3	MSCCCV	1	MSCCCV
S8	4.855	1	MSCCCV	1	MSCCCV

In order to get the ground truth SOH for each battery cycled in the laboratory, a low current probe cycle was repeated after every 50 cycles. In the probe cycle, batteries were CCCV charged and CC discharged at 0.2C. The capacity of a probe cycle was computed by coulomb counting.

5 $$\begin{aligned} C_{probe} = \int _{0}^{T}I_{probe} dt, \end{aligned}$$

where $C_{probe}$ and $I_{probe}$ stand for capacity and current of the probe cycle respectively. SOH was computed from capacity using (1). As probe cycles were done after every 50 cycles, linear interpolation was used to obtain the SOH of intermediate cycles. Let $SOH_c$ and $SOH_{c+50}$ be the SOH values computed using probe at cycle number *c* and $c+50$ respectively. SOH of intermediate cycles were obtained by sampling the straight line joining $(c, SOH_c)$ and $(c+50, SOH_{c+50})$.

6 $$\begin{aligned} SOH_{c+i} = SOH_{c}+(c+i-c)\left(\frac{SOH_{c+50}-SOH_c}{c+50-c}\right), \end{aligned}$$

where $SOH_{c+i}$ denotes the SOH for cycle number $c+i$, and $1<i<49$.

**Figure 9:**
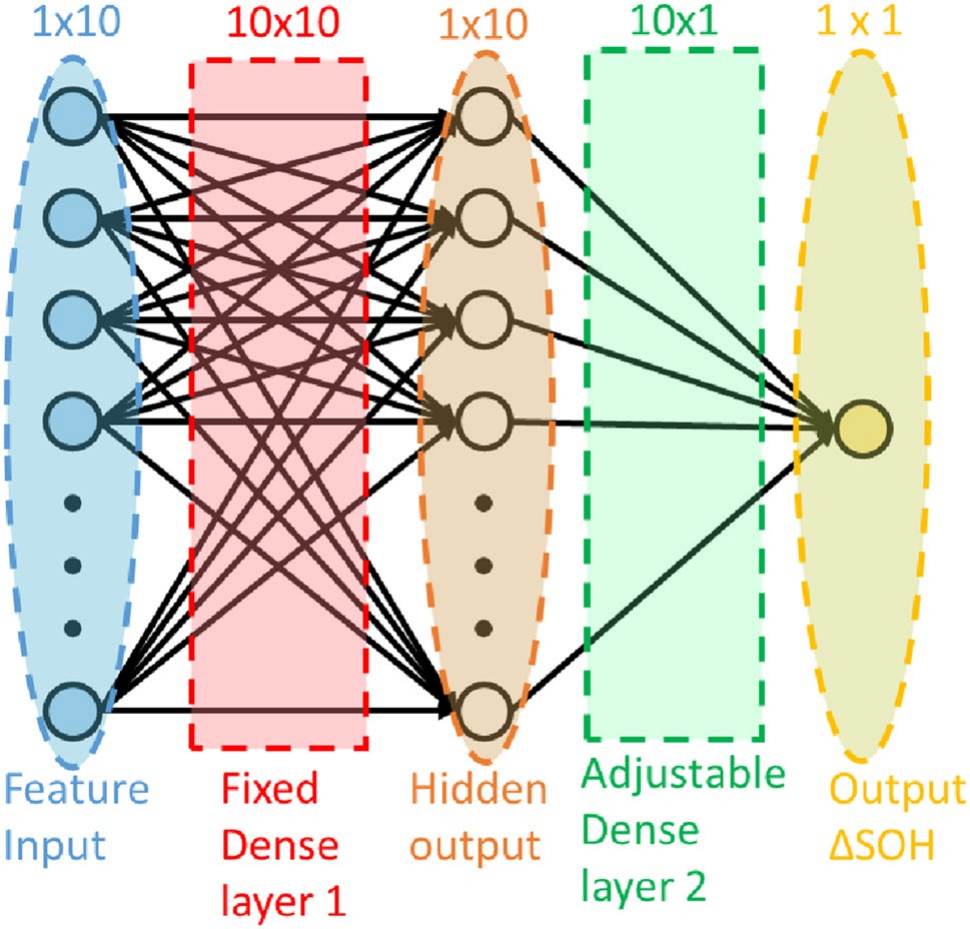
The two layer ANN used for mapping $\Delta V_r$ to $\Delta SOH$.

**Figure 10:**
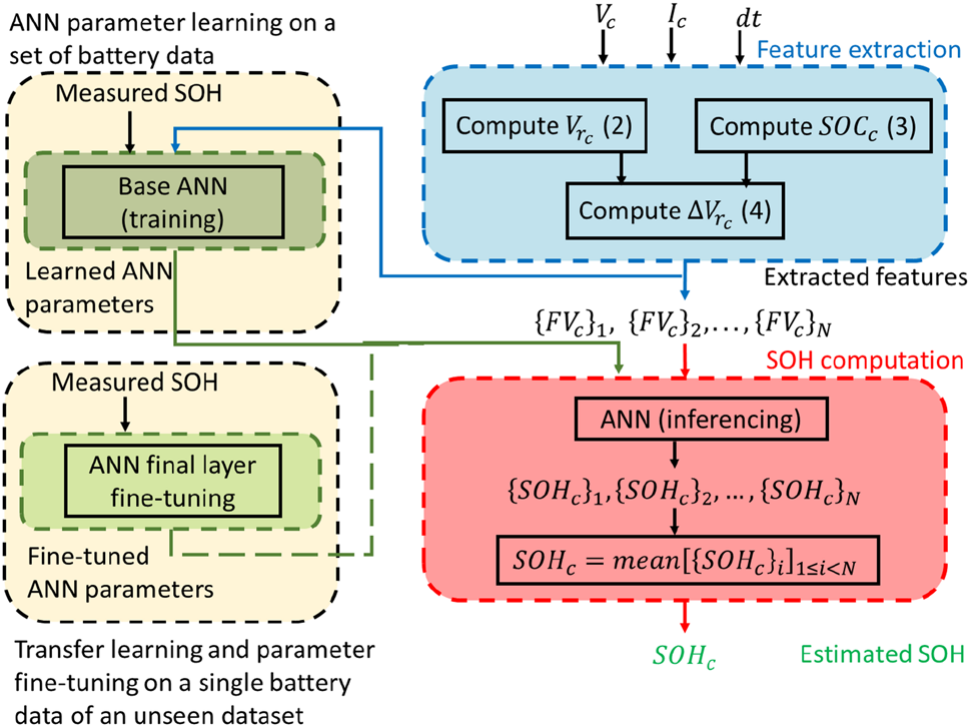
Flow chart of the method.

### ANN used for SOH mapping

A simple two-layer ANN has been used to model the relation between $\Delta V_r$ and $\Delta SOH$. The feature label relation is largely linear (Fig. 3). We have observed through experimental validation that the ANN outperforms linear regression model when used on batteries of unseen devices. Additionally, transfer learning is possible using ANNs, where, the model generalizes on new data while retaining its earlier learning. The ANN has been shown in Fig. 9. As shown in the figure, input to the ANN has dimension 1x10. A hidden dense layer of 10x10 has been used which takes a 1 x 10 input and produces a hidden output of dimension 1 x 10. Then, a second dense layer of 10x1 takes the hidden layer output as input and computes the SOH value. Ten consecutive samples of $\Delta V_r$ computed at a gap of 2% SOC constitutes one feature vector. A set of feature vectors computed in the SOC range 30–50% at different SOH values have been shown in (d), (h), and (l) of Fig. 2. One feature vector can be computed for every 20% SOC range. The ANN takes one $\Delta V_r$ feature vector as input and gives $\Delta SOH$ as output. Multiple SOH values are computed using feature vectors from multiple SOC windows in the 20–90% SOC range. The mean of all SOH values is considered as the SOH of the cycle.

### SOH estimation method

A flowchart of the steps followed in the proposed SOH estimation method has been depicted in Fig. 10. The method primarily consists of two steps: offline training or fine-tuning of ANN, and online estimation of SOH.

#### Feature extraction

Following are the steps to compute feature vectors from the measured voltage and current in a particular cycle.

1. *SOC* is computed using current and sampling interval *dt* as shown in (3). $V_r$ is computed using voltage and current as depicted in (2).
2. $V_{r_0}$ and $SOC_0$ of the first cycle of the fresh battery are saved for computation of $\Delta V_{r}$ in later cycles.
3. $\Delta V_{r_c}$ for a cycle *c* is computed using (4) for $20\%\le SOC<90\%$.
4. Feature vectors (*FV*) are formed using 10 samples of $\Delta V_{r_c}$ sampled at an *SOC* interval of 2%. For eg. *FV* for $30\%\le SOC<50\%$ is

7 $$\begin{aligned} \begin{aligned} FV_{30\%\le SOC<50\%} = [&\{\Delta V_{r_c}\}_{SOC=30\%}, \{\Delta V_{r_c}\}_{SOC=32\%},&..., \{\Delta V_{r_c}\}_{SOC=48\%}]. \end{aligned} \end{aligned}$$

The next *FV* is computed for $31\%\le SOC<51\%$. The complete *FV* set for $20\%\le SOC<90\%$ is $\{FV_{20\%\le SOC<40\%}, \; FV_{21\%\le SOC<41\%}, \; ..., FV_{71\%\le SOC<89\%}\}$.

#### Training of base ANN

The base ANN model was trained using data from multiple batteries. One cell from each set listed in Table 6 was chosen to train the ANN. Cells were randomly selected from each set. Feature vectors were computed as described in previous section. Total 62512 feature vectors were extracted from the charging data of those 8 cells. $\Delta SOH$ labels for the *FV*s were computed from the ground truth SOH values obtained using probe cycles.

The extracted feature and label set were randomly split into 80:20 ratio for training and validation. A windows machine having Intel core i7 processor was used for training. Code was written in the PyTorch framework. The training was run for 50 epochs with Adam optimizer and L1 loss function. A learning rate of 0.01 was used. The model was tested on the validation set after every epoch. The best model with minimum loss was saved.

#### Transfer learning for new dataset

The feature label proportionality relation will change when battery chemistry or physical dimension change^24,25^. The ANN model has to be fine-tuned to adapt to the new dataset. The first layer of the base ANN is kept unchanged. The second layer is fine-tuned using only one cell data of the new set. Same training hyper parameters such as loss function, learning rate, number of epochs, optimizer, etc. were used for model tuning.

#### SOH estimation of an unseen battery

Cells that were excluded during training or fine-tuning were used for blind testing of the learned ANN model. Following are the steps to estimate SOH from charging data of a particular cycle.

1. Feature vectors are computed from charging voltage and current in a cycle *c*.
2. All feature vectors are passed through ANN to get $\Delta SOH$ values.
3. Mean of all $\Delta SOH$ values is considered as $\Delta SOH$ for the cycle.

### Evaluation metrics

For quantitative evaluation of the proposed SOH estimation method, following parameters have been used.

8 $$\begin{aligned}&MAE = \sum _{c=1}^{c=N}\mid \{SOH_c\}_{actual}-\{SOH_c\}_{predicted}\mid ; \end{aligned}$$

9 $$\begin{aligned}{}&RMSE = \sqrt{\dfrac{1}{N}\sum _{c=1}^{c=N} \left( \{SOH_c\}_{actual}-\{SOH_c\}_{predicted}\right) ^2}; \end{aligned}$$

10 $$\begin{aligned}{}&SDE = \sqrt{\dfrac{1}{N}\sum _{c=1}^{c=N}\left( Error_c-\overline{Error}\right) ^2}; \end{aligned}$$

where, N is the total number of cycles in a single battery data. $\{SOH_c\}_{actual}$ and $\{SOH_c\}_{predicted}$ are the actual and predicted SOH values for cycle *c*. $Error_c=\{SOH_c\}_{actual}-\{SOH_c\}_{predicted}$, and $\overline{Error}=\dfrac{1}{N}\sum _{c=1}^{c=N}Error_c$.
